# Common Recruitment of Angular Gyrus in Episodic Autobiographical Memory and Bodily Self-Consciousness

**DOI:** 10.3389/fnbeh.2018.00270

**Published:** 2018-11-14

**Authors:** Lucie Bréchet, Petr Grivaz, Baptiste Gauthier, Olaf Blanke

**Affiliations:** ^1^Laboratory of Cognitive Neuroscience, Brain Mind Institute, School of Life Sciences, Swiss Federal Institute of Technology (EPFL), Campus Biotech, Geneva, Switzerland; ^2^Center for Neuroprosthetics, Swiss Federal Institute of Technology (EPFL), Campus Biotech, Geneva, Switzerland; ^3^Department of Neurology, Geneva University Hospital, Geneva, Switzerland

**Keywords:** multisensory integration, parietal cortex, bodily self-consciousness, out-of-body experience, episodic autobiographical memory, ALE meta-analysis, fMRI, lesion analysis

## Abstract

Parietal cortex and adjacent parts of the temporal cortex have recently been associated with bodily self-consciousness (BSC). Similarly, growing evidence suggests that the lateral parietal cortex is crucial for the subjective aspects of episodic autobiographical memory (EAM), which is based on the conscious experience of reliving past events. However, the neuroanatomical relationship between both fundamental aspects remains currently unexplored. Moreover, despite the wealth of neuroimaging data on EAM, only few neuroimaging studies have examined BSC and even fewer examined those aspects of BSC that are most closely related to EAM. Here, we investigated whether regions in the inferior parietal lobule (IPL) that have been involved in spatial aspects of BSC (self-location and first-person perspective), as described by Ionta et al. (2011) are also active in studies investigating autobiographical memory. To examine this relation, we thus compared the regions indicated in the study by Ionta et al. (2011) based on data in healthy participants and neurological patients, with the results from a meta-analytical study we performed based on functional neuroimaging studies on EAM and semantic autobiographical memory (SAM). We report an anatomical overlap bilaterally in the angular gyrus (AG), but not in other parietal or temporal lobe structures between BSC and EAM. Moreover, there was no overlap between BSC and SAM. These preliminary data suggest that the bilateral AG may be a key structure for the conscious re-experiencing of past life episodes (EAM) and the conscious on-line experience of being located and experiencing the world in first-person (BSC).

## Introduction

The subjective feeling of a unified self that is experienced as residing in one’s own body, which is localized at a specific position (self-location) and from where one perceives the world (first-person perspective) defines the three major components of multisensory bodily self-consciousness (BSC; Blanke and Metzinger, 2009; Blanke et al., 2015). Although the experimental investigation of BSC and the underlying brain networks remains a challenge, recent advances in digital technologies have provided compelling ways of successfully inducing illusory states of BSC in healthy individuals by manipulating multisensory bodily cues (Ehrsson, 2007; Lenggenhager et al., 2007; Petkova and Ehrsson, 2008). Recently, illusory self-location and first-person perspective were manipulated in a fMRI study (Ionta et al., 2011) by applying tactile stroking to the back of a participant, while simultaneously displaying the stroking on the back of a virtual body via a head mounted display. Such experimentally-induced changes in BSC have been linked to activity at the temporo-parietal junction (TPJ; Brodmann area 39/40). Likewise, lesions, seizures or electrical brain stimulation in the TPJ area also result in changes in self-location and first-person perspective (Blanke et al., 2002, 2004; De Ridder et al., 2007; Ionta et al., 2011). Thus, patients suffering from so-called out of body experiences (OBE) caused by brain damage at the TPJ, subjectively experience the world from an illusory disembodied self-location with an inverted direction of first-person perspective and they self-identify with this elevated position (Blanke and Arzy, 2005; Ionta et al., 2011; Heydrich and Blanke, 2013).

The subjective sense of self in time that enables us to re-experience ourselves in the past and mentally project ourselves into the future, i.e., autonoetic consciousness, has been considered the defining aspect of episodic autobiographical memory (EAM) recollection (Tulving, 1985). Traditionally, since the discovery of severely amnesic patients with damage to the medial temporal lobe (MTL; Scoville and Milner, 1957; Steinvorth et al., 2005), many fMRI studies confirmed the essential role of MTL structures in memory (Svoboda et al., 2006; Cabeza and St. Jacques, 2007; Daselaar et al., 2008; St. Jacques et al., 2015; Clark and Maguire, 2016). Relevant to the present study, numerous neuroimaging studies on episodic memory (Cabeza and St. Jacques, 2007; Sestieri et al., 2011, 2017; Rugg and Vilberg, 2013; St. Jacques et al., 2013; Gilmore et al., 2015; Bellana et al., 2017; Rutishauser et al., 2017) also reported persistent and robust activations in the lateral parietal cortex, particularly the angular gyrus (AG; Brodmann area 39). Interestingly, patients with lateral parietal lesions are successful in objective EAM tasks, however, it has been found that the vividness, richness and subjective confidence in experiencing their personal memories is diminished (Berryhill et al., 2007; Simons et al., 2010; Hower et al., 2014; Rugg and King, 2018). These neuropsychological, fMRI and TMS studies suggest that the sole engagement of MTL structures may not be sufficient for the multi-modal and conscious experiences, which accompany subjective, detail-rich and self-related EAM recollection, and that additional regions, such as those in the lateral parietal cortex, are involved as well.

The activity in the AG has also been associated with semantic memory (Binder et al., 2009; Seghier et al., 2010; Bonnici et al., 2016; Humphreys and Lambon Ralph, 2017). Recent resting state function connectivity MRI studies proposed that the lateral parietal cortex, particularly the AG, may represent a heterogeneous area comprised of functionally and anatomically distinct sub-regions (Nelson et al., 2010, 2013; Daselaar et al., 2013; Seghier, 2013), also questioning whether AG supports the same processes during episodic and semantic retrieval. Critically, Brown et al. (2018) demonstrated that the left posterior parietal cortex contributes to both EAM and semantic autobiographical memory (SAM), but each demonstrating divergent activities. More specifically, the AG exhibited a graded pattern of activity, declining from episodic recollection to correct rejections of novel events in semantic remembering. Consistent with the prior literature, we therefore hypothesized that SAM (i.e., the general self-awareness of personal facts), which is independent of re-experiencing particular, conscious, vivid and multi-modal life episodes, would not anatomically overlap with BSC activations in lateral parietal cortex.

Given the link of BSC with subjective experience and seminal proposals by Endel Tulving that subjective re-experiencing of specific past events is a fundamental component of EAM (Tulving, 1985, 2005), BSC and autonoetic consciousness may share neural mechanisms. As subjective re-experiencing of own life events is often characterized by a viewpoint and location from where the event is re-experienced, it may be argued that the two spatial components of BSC (first-person perspective and self-location) are of particular relevance for EAM. Moreover, St. Jacques et al. (2017) showed (using different methodology) that first-vs. third-person perspective during memory retrieval modulated recall of autobiographical events, associated with lateral parietal activations, potentially overlapping with BSC activations. Despite several reviews discussing the role of parietal lobe in BSC (Blanke, 2012; Serino et al., 2013; Blanke et al., 2015) as well as the recent interest in the contribution of parietal lobe to EAM (Moscovitch et al., 2016; Igelström and Graziano, 2017), it is currently unknown whether and to what extent BSC and autobiographical memories (episodic or semantic) engage the same or distinct brain regions. In order to provide preliminary data, we investigated the question of overlap between the spatial aspects of BSC and EAM by studying whether the activations in inferior parietal lobule (IPL) and the adjacent parts of the posterior temporal cortex during the experimental manipulation of both aspects of BSC, as observed in the study by Ionta et al. (2011) in healthy subjects, overlap with those described in EAM studies. We further included results from a lesion analysis study of nine neurological patients with OBEs (abnormal self-location and abnormal first-person perspective) whose brain damage was also localized at TPJ. We performed a systematic quantitative coordinate based meta-analysis (Eickhoff et al., 2009, 2012) on human EAM as well as SAM functional neuroimaging studies to examine whether and where brain regions associated with autobiographical memories share common or distinct neural substrates with BSC as reported in the study by Ionta et al. (2011). Based on the evidence that both EAM and BSC recruit the lateral parietal cortex and given the fundamental link between BSC and EAM with multi-modal subjective and conscious experiences, we hypothesized that there would be an anatomical overlap in this structure between BSC and autobiographical memory, specifically for the episodic (EAM), but not the semantic aspects (SAM). Consistent with the prior literature, EAM would be related to BSC in the posterior parietal cortex, particularly the AG region, because of its role in integrating cross-modal, multi-sensory features to form a unified episodic memory representation, which is fundamentally related to multi-sensory BSC signals (i.e., particularly the first-person perspective and self-location).

## Materials and Methods

### Experimental and Clinical Studies on Bodily Self-Consciousness (BSC)

We evaluated data from our previously performed fMRI study in 22 healthy subjects (*M* = 25.4, SD = 5.7, 22 males), assessing neural mechanisms of BSC using multisensory stimulation. In this study, Ionta et al. (2011) experimentally manipulated two global aspects of BSC (self-location and first-person perspective) by using an MRI-compatible robot. Based on the original study by Lenggenhager et al. (2007), which used virtual reality technology (VR) to experimentally manipulate BSC, (Ionta et al., 2011) applied synchronous tactile stimulation to the participants’ back while lying in the MRI scanner. Participants observed a full body avatar being stroked congruently on its back from a third-person perspective. After synchronous visuo-tactile stimulation, participants showed higher self-identification towards the virtual body, as compared to the asynchronous condition. Furthermore, about half of the participants experienced an upward looking first-person perspective (compatible with the physical orientation of their body), while the remaining half of the participants had the impression to be looking down (down-ward-looking perspective). This perspective was incompatible with the orientation of their body, compatible with a third-person perspective induced by the full-body illusion. Moreover, the study by Ionta et al. (2011) also included results from lesion analysis of nine neurological patients, suffering from a carefully defined altered state of BSC, namely out-of-body experiences (OBEs) caused by focal brain damage (OBEs are characterized by abnormal self-location and abnormal first-person perspective). By normalizing each patient’s lesion into common reference space, statistical lesion overlap comparison was carried out, contrasting the lesions of the OBE-patients with those from a control group using voxel-based lesion symptom mapping (VLSM; Bates et al., 2003). For more details, see Supplementary information in Ionta et al. (2011).

### Selection of Studies and Inclusion Criteria for EAM and SAM Meta-Analyses

For both the EAM as well as the SAM studies, we conducted a comprehensive and systematic search of the literature using PubMed^1^ and Web of Knowledge^2^. The following combination of keywords was used for EAM: “autobiographical memory,” “episodic.” For the SAM, we selected: “autobiographical memory,” “semantic.” The reference lists of the included studies and several previous meta-analyses were used to find studies (Svoboda et al., 2006; Martinelli et al., 2013; Kim, 2016). Studies using positron emission tomography (PET) or functional magnetic resonance imaging (fMRI) were included in the analyses. Studies were considered only if activation coordinates were reported in standardized Montreal Neurological Institute (MNI) or Talairach (TAL) coordinates and the analysis reported on the whole brain. All TAL coordinates were transformed into the MNI coordinates using a linear transformation (Lancaster et al., 2007).

Only study results in healthy subjects with no neurological or psychiatric disorders, brain lesions or pharmacological manipulations were considered. Studies including both younger and older healthy participants were included in the analysis. No single subject studies were considered for the analyses. If articles reported several experiments with independent samples, then these experiments were considered individually in the analysis. Visual as well as auditory cues were included irrespectively of their emotional valence. Both recent and remote memories for the EAM analysis were included. Personal events and judgments of the self-vs. others were included in the SAM analysis.

#### Episodic (EAM) and Semantic (SAM) Autobiographical Memory Studies

Forty-one experiments investigated EAM (Supplementary Table S1) and this meta-analysis included 588 foci and 813 subjects. We focused primarily on studies investigating self-related, personally-relevant EAM irrespective of their specificity, age or control task. Thus, we included both recent and remote EAM (e.g., Oddo et al., 2010; Addis et al., 2012), using semantic memory (Donix et al., 2010; Holland et al., 2011) as well as low level baseline condition (e.g., rest or pseudo words; Nadel and Moscovitch, 1997; Piolino et al., 2008) as control tasks. We selected 25 experiments examining SAM (Supplementary Table S2) and included 314 foci and 396 subjects. Particularly, we included studies investigating familiar, self-relevant semantic information (e.g., faces, places, objects), but also self-trait judgments (Sugiura et al., 2009, 2011). Control task in the SAM category included unfamiliar information or other-trait judgments (e.g., Gutchess et al., 2007; Jenkins et al., 2008).

### Data Analysis for EAM and SAM Studies

We employed the quantitative activation likelihood estimation (ALE) algorithm as implemented in the GingerALE software, v2.3.6 (Eickhoff et al., 2009, 2012; Turkeltaub et al., 2012). The ALE algorithm ultimately aims at assessing statistically whether a specific task activates each specific voxel of the brain more likely than by chance (Eickhoff et al., 2009). To account for spatial uncertainty in the location of activity, each voxel at the location of the peak-activation from each contrast and experiment is convolved with a 3-dimensional Gaussian kernel whose full-width at half maximum is weighted by the number of subjects used in that particular experiment. The sum of these Gaussians constitute the modeled activation (MA) maps. We used a within-cluster *p* < 0.05 FWE correction with *p* < 0.001 uncorrected as the cluster-forming threshold. The minimal cluster size was set to 200 mm^3^. Visualization of foci and the activation clusters from the above mentioned analyses was performed using MRIcron software^3^ and the clusters were labeled using the Automated Anatomical Labeling (AAL) atlas (Tzourio-Mazoyer et al., 2002) and Brodmann atlas implemented within the same software.

## Results

### EAM Regions (Individual ALE Analysis)

The ALE meta-analysis of the 41 EAM studies uncovered 10 clusters (Table 1). More specifically, we found activation bilaterally in the AG (Brodmann area 39) and the left superior temporal gyrus (STG, Brodmann area 22). We found consistent activations in cortical midline structures, i.e., bilaterally in the ventral and dorsal parts of the posterior cingulate cortex (PCC; Brodmann areas 31 and 23) and left anterior cingulate cortex (ACC, Brodmann area 10). The analysis also revealed activity bilaterally in the hippocampi and adjacent parahippocampal gyri (PHG; Brodmann areas 28, 35, 36) and in the left inferior temporal gyrus (ITG, Brodmann area 21). Other clusters were found in the left inferior frontal gyrus (IFG, Brodmann area 47), bilaterally in superior frontal gyrus (SFG, Brodmann area 32) and in the left middle frontal gyrus (MFG, Brodmann area 6).

**Table 1:** Results from episodic autobiographical memory (EAM) meta-analysis.

Brain region	Hemisphere	Size (mm^3^)	*x*	*y*	*z*	Brodmann area
Angular gyrus (TPJ)	L	3,696	−48	−62	22	39
Angular gyrus (TPJ)	R	1,040	60	−60	20	39, 22
Posterior cingulate cortex	B	10,248	−4	−58	26	31, 23
Anterior cingulate cortex	L	2,536	−4	58	−12	10
Parahippocampal gyrus	L	10,528	−24	−16	−20	28, 36
Parahippocampal gyrus	R	6,200	20	−20	−16	35, 36
Inferior temporal gyrus	L	2,176	−62	−8	−20	21
Inferior frontal gyrus	L	3,072	−36	24	−12	47
Superior frontal gyrus	B	2,248	8	20	52	32
Middle frontal gyrus	L	1,176	−40	8	42	6

*Within-cluster FWE-corrected *p* < 0.05 with *p* < 0.001 (uncorrected) as the cluster forming threshold. R, right; L, left; B, bilateral; x, y, z are the coordinates in MNI space. Label provided using the Brodmann atlas*.

### SAM Regions (Individual ALE Analysis)

The ALE meta-analysis of the 25 SAM studies uncovered two clusters (Table 2). These were located in the cortical midline structures, i.e., bilateral ventral and dorsal parts of the ACC (Brodmann areas 32, 24) and bilaterally in the ventral and dorsal PCC (Brodmann area 31 and 23). There was no activity in the region of the angular gyri.

**Table 2:** Results from semantic autobiographical memory (SAM) meta-analysis.

Brain region	Hemisphere	Size (mm^3^)	*x*	*y*	*z*	Brodmann area
Anterior cingulate cortex	B	5,032	−6	44	4	32, 24
Posterior cingulate cortex	B	1,504	2	−60	20	31, 23

*Within-cluster FWE-corrected *p* < 0.05 with *p* < 0.001 (uncorrected) as the cluster forming threshold. R, right; L, left; B, bilateral; x, y, z are the coordinates in MNI space. Label provided using the Brodmann atlas*.

### Anatomical Overlap Between BSC, EAM and SAM

The BSC regions from the fMRI study on healthy subjects by Ionta et al. (2011) were located at the left and right TPJ and included the posterior part of the superior temporal gyrus, the parietal operculum, the posterior insula and superior portion of the supramarginal gyrus (lTPJ MNI: −54, −32, 20; rTPJ MNI: 55, −28, 16). The quantitative lesion analysis of the patient data in Ionta et al. (2011) revealed a maximal lesion overlap at the rTPJ and included the posterior end of the superior and middle temporal and angular gyri (MNI: 54, −52, 26), which was located somewhat posterior to the fMRI-based BSC regions. The union of the regions from the fMRI and lesion analyses defined our BSC regions (Table 3).

**Table 3:** Results from bodily self-consciousness (BSC) experimental study (*N* = 22) FDR-corrected, *p* < 0.05 and neurological patients study (*N* = 9) based on voxel-based lesion symptom mapping (VLSM) FDR-corrected, *p* < 0.01.

Brain region	Hemisphere	Size (mm^3^)	*x*	*y*	*z*	Brodmann area
Superior temporal gyrus	L	192*	−54	−32	20	39, 40
Superior temporal gyrus	R	197*	55	−28	16	39, 40
Temporal parietal junction	R		54	−52	26	39, 40

R, right; L, left; B, bilateral; x, y, z are the coordinates in MNI space. Label provided using the Brodmann atlas. *Active cluster size in voxels.

**Figure 1:**
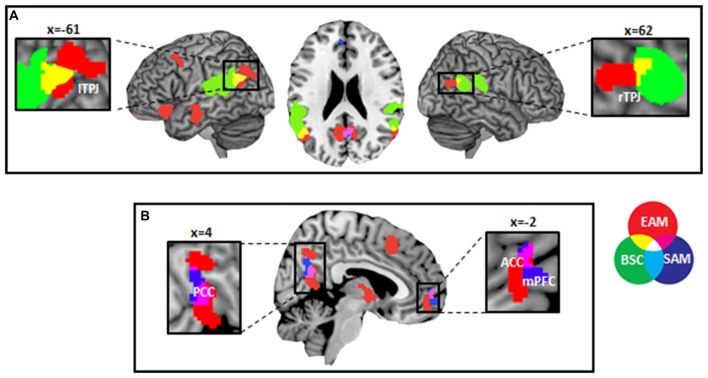
Results of overlap analysis. **(A)** Visualization of the anatomical overlap between bodily self-consciousness (BSC; Ionta et al., 2011) and episodic autobiographical memory (EAM) activation likelihood estimation (ALE) analysis in bilateral parietal cortex. **(B)** Visualization of the anatomical overlap between semantic autobiographical memory (SAM) and EAM ALE analyses in the cortical midline structures. Within-cluster FWE-corrected *p* < 0.05 with *p* < 0.001 (uncorrected) as the cluster forming threshold. Label provided using the MRIcron.

We found an anatomical overlap between EAM regions and BSC regions bilaterally in the AG (Brodmann area 39; Figure 1A). The size of the anatomical overlap between BSC and EAM was 1,192 mm^3^ on the left side and 128 mm^3^ on the right side. EAM and SAM overlapped in the ventral and dorsal parts of the PCC (Brodmann areas 31 and 23) and bilaterally in the dorsal part of ACC (Brodmann area 32; Figure 1B). The size of the anatomical overlap between the ventral and dorsal parts of the PCC was 776 mm^3^ and 560 mm^3^ in the dorsal part of the ACC.

## Discussion

The present study extends previous EAM studies by investigating the role of subjective, conscious processing in autobiographical memory, based on spatial aspects of BSC (self-location and first-person perspective). Given the link of BSC with subjective experience and seminal proposals by Endel Tulving that subjective re-experiencing of specific past events is a fundamental component of EAM (Tulving, 1985, 2005), we speculated that neural processes related to multisensory bodily processing are not only relevant for BSC, but also for consciousness concerning past events and EAM (i.e., autonoetic consciousness). Subjective re-experiencing of own life events is often described as if experiencing the remembered event from a viewpoint and location that is similar to that during encoding of the event. Accordingly, we speculated that the first-person perspective and self-location of BSC are of particular relevance for autonoetic consciousness and EAM. The current data are preliminary (as we could only include data from Ionta et al., 2011), but allow to confirm this prediction. More brain imaging data on these two spatial aspects of BSC is needed to further confirm our findings.

Our findings suggest that EAM activity indeed anatomically overlaps with BSC in the AG. Previous work on BSC investigated self-related processing by using multisensory cues through a variety of experimental paradigms, highlighting contributions from own body signals (Ehrsson, 2007; Blanke and Metzinger, 2009; Tsakiris et al., 2010; Blanke, 2012; Serino et al., 2013; Bergouignan et al., 2014; Blanke et al., 2015). We note that during the encoding of personal-life episodes, multisensory signals about the body of the observer that are of relevance for BSC are always perceptually co-present. Such signals are thus potentially of relevance for BSC and EAM, even though during encoding these signals often remain in the background. For instance, (Ciaramelli et al., 2010) found that patients with left-lateralized lesions in the posterior parietal cortex suffered from an impaired and disembodied, subjective experience associated with a navigation task. Moreover, Bergouignan et al. (2014) reported that recall of EAM items and hippocampal activity during the encoding of episodic events is modulated by the visual perspective from where the event was viewed during encoding. Using different methodology, St. Jacques et al. (2017) showed that first-vs. third-person perspective during memory retrieval modulated recall of autobiographical events, and associated this with medial and lateral parietal activations. In a recent study (Bréchet et al., 2018), VR technology has allowed us to experimentally control and manipulate key elements of BSC during EAM encoding and retrieval, revealing that classical BSC factors also influence EAM performance. Thus, our current findings are compatible with these data on shared resources for BSC and EAM and reveal an anatomical overlap bilaterally at the level of AG. However, many different manipulations of perspective and visual viewpoints have been carried out in the past (i.e., see Blanke, 2012). The subjective and behavioral changes as well as the associated brain activity as investigated for example by Ionta et al. (2011) or St. Jacques et al. (2017) are thus not easily comparable. Thus, more work is also needed to investigate the common and distinct brain mechanisms involved in these different “perspective” manipulations.

Our finding is in line with previous neuropsychological and neuroimaging studies (Svoboda et al., 2006; Kim et al., 2012; Foster et al., 2015; Kim, 2016), consistently reporting the lateral parietal region as the second most frequently activated region outside MTL structures during EAM retrieval. Although the lateral parietal cortex has been often associated with visuo-spatial perception, attention (Corbetta and Shulman, 2002) and multisensory integration (Driver and Noesselt, 2008; Tomasino and Gremese, 2016), it is only more recently that studies associated the lateral parietal cortex and especially the AG with retrieval related to EAM (Cabeza and St. Jacques, 2007; Sestieri et al., 2011, 2017; Rugg and Vilberg, 2013; Gilmore et al., 2015; Moscovitch et al., 2016; Bellana et al., 2017; Igelström and Graziano, 2017; Rutishauser et al., 2017). This has led to several suggestions about the contributions of the parietal cortex, especially AG, to EAM retrieval. For example, the working buffer hypothesis (Vilberg and Rugg, 2008) suggested different roles for the ventral and dorsal regions in lateral parietal cortex, linking ventral parietal cortex to EAM recollection (i.e., the ability to subjectively re-experience past events enabled by autonoetic consciousness) and the dorsal parietal cortex to familiarity related processing (i.e., general recognition of events without any details associated with noetic consciousness; Cabeza et al., 2008). Recently, Bonnici et al. (2016) investigated the role of the AG during retrieval of unimodal and multimodal episodic and semantic memories. Their findings suggest that the AG may enable the multimodal (i.e., audio-visual) integration of sensory features into rich, vivid and subjectively relevant EAM. This interpretation of the essential role of AG in the subjective experiencing of multi-sensory past events is consistent with the recent research on BSC that highlighted the multi-sensory and sensorimotor processing and integration of different bodily stimuli to the sense of self localized in the lateral parietal area (i.e., Blanke et al., 2015). Nonetheless, the precise role of AG in memory and autobiographical memory is still a matter of controversy.

The current study revealed bilateral lateral posterior parietal activations in the AG (Brodmann area 39) for EAM, but not SAM. There is an increasing debate whether the neural correlates of EAM and SAM are shared or distinct. While Levine et al. (2004) claim distinct neural correlates for EAM and SAM, Renoult et al. (2012) suggest that the neural correlates of SAM may closely resemble episodic or semantic depending on the memory measures. Specifically, autobiographical fact and self-knowledge, which are heavily involved in self-referential processing, activate the MPFC. This finding is in line with a recent meta-analysis of Martinelli et al. (2013), which showed that SAM and conceptual self (CS) activated the medial prefrontal structures. Confirming our finding of ACC and PCC activity for SAM, recent meta-analyses that involved trait-judgments of the self vs. other or self vs. others events, suggested a critical role of anteromedial prefrontal cortex and posterior cingulate cortex in the self-referential processing (Northoff et al., 2006; Qin and Northoff, 2011; Kim, 2012). For example, the meta-analyses of Kim (2012) suggested that conscious memory retrieval, both semantic and episodic, is associated with the involvement of the intrinsic default mode network (DMN). Importantly, Piolino et al. (2009) emphasized that the lifelong changes in autobiographical memory play a critical role in the debate about the neural basis of EAM and SAM. Indeed, it is important to keep in mind the well-known temporal shift of autobiographical memories from episodic to semantic (Conway et al., 1997). This temporal shift corresponds to the concept of semantization of long-term memories over time (Brewer, 1986), which suggests that long-term autobiographical memories become a combination of both subjective experiences of EAM and self-knowledge and facts of SAM (Westmacott and Moscovitch, 2003; Cabeza and St. Jacques, 2007; Piolino et al., 2007). Similarly, it is relevant to note the aspect of memory repetition, which also influences the autobiographical recollection. While re-living similar episodes (the so-called external repetition; for more detail see Piolino et al., 2009) may lead to decontextualization of past events, thinking or talking about the past event (the so-called internal repetition) may enhance the details of the past events (Rubin and Kozin, 1984; Nadel et al., 2007). Renoult et al. (2012) suggested an intermediate category of declarative memory, the so-called personal semantics (i.e., autobiographical facts, such as “Jacob is the name of my brother”), which he compares to the general semantic knowledge about the world (such as “Jacob was the son of Isaac in the Old Testament”). The authors pointed out that neuropsychological studies using Autobiographical Memory Interview (AIM) of Kopelman et al. (1989) generally assume the same neural correlates of both personal and general semantics. Interestingly, several studies of Maguire and colleagues (Maguire and Mummery, 1999; Maguire and Frith, 2003) suggested an overlapping activity in medial prefrontal region for both personal and general semantics, however personal semantics showed stronger, left-lateralized activity in prefrontal cortex, retrosplenial cortex, temporal pole and temporoparietal junction. It is important to note that our current meta-analysis on SAM did not distinguish between studies investigating familiar, self-relevant semantic information (e.g., faces, places) and studies examining self-trait judgments. We highlight that our finding of CMS for SAM meta-analysis is similar to previous studies on self-referential processing, however we cannot exclude the possibility that separating the self-referential judgments as an independent meta-analysis could have led to further findings. The inclusion of incongruent studies is an important limitation of the present meta-analysis on autobiographical memory. Future work should specifically focus on particular aspects of episodic and SAM, including the personal semantic and general semantic memory and also improve the analysis with respect to BSC.

Tulving (1972) theoretically differentiated two main components of autobiographical memory by their distinct states of consciousness. SAM has been characterized by the noetic consciousness (i.e., subjects remember personally relevant facts; Tulving, 2002; Schacter et al., 2007). Contrary, autonoetic consciousness defines the EAM (i.e., subjects re-experience the past or project themselves into the future; Tulving, 1972). Based on the evidence that both EAM and BSC recruit the lateral parietal cortex and given the fundamental link between BSC and EAM with multimodal subjective, conscious experiences, we hypothesized that there would be an anatomical overlap in this structure between BSC and autobiographical memory, specifically for the episodic (EAM), but not the semantic aspects (SAM). The present study confirms and extends previous EAM studies, by showing that the neural processes related to multisensory bodily processing are not only relevant for BSC, but also for consciousness concerning past events and EAM (i.e., autonoetic consciousness). Thus, the present ALE data on EAM and SAM corroborate the recent proposals about the involvement of the lateral parietal cortex, including the AG and adjacent regions of the superior temporal gyrus in EAM. Accruing evidence points toward the causal role of AG in the subjective, conscious recollection of past episodes. The current findings are thus consistent with the proposal that particularly the AG and not the cortical midline structures that are involved in the self-referential processing, is involved in integrating multiple memory features into a multi-modal (i.e., including sounds, smells, sights) conscious representation that enables the rich and vivid subjective re-living of an event.

The present data suggest that AG (revealed by the study of Ionta et al., 2011) was jointly involved in BSC and EAM. Unlike neurological patients with damage to MTL structures (Scoville and Milner, 1957; Steinvorth et al., 2005), patients with lateral parietal lobe damage do not suffer from severe EAM deficits. For this reason, the subtler EAM impairments associated with damage to the lateral parietal cortex were previously largely overlooked. However, recent clinical studies investigating memory in patients with parietal lesions have provided valuable insights and especially highlighted contributions of the AG to EAM (Berryhill et al., 2007; Simons et al., 2010; Hower et al., 2014). Even though patients with damage to parietal cortex are able to retrieve past personal events, a number of such lesion studies revealed that the ventral part of the lateral parietal cortex, especially the left AG, is associated with the impairment of subjective re-experiencing of vivid, rich and multi-sensory EAM. Moreover, patients with parietal lobe damage often report lower confidence in the retrieval of their EAM. These findings suggest a particular role of AG in the subjective experience of EAM recollection and indicate that the MTL may not be sufficient for the full-blown subjective experience of the self in the past. Critically, it is important to mention a number of neuroimaging studies that have described the functional differentiation on the level of neural networks, which play a role in episodic memory retrieval, particularly the MTL structures and frontoparietal regions. These neuroimaging studies demonstrated that an activation within these latter brain circuits often reflects the memory recollection vs. the subjective feeling of familiarity related to the contextual memory details (Squire et al., 2004; Eichenbaum et al., 2007; Rugg and Vilberg, 2013). This functional heterogeneity within parietal regions and between the sub-regions of MTL activity patterns supports the different aspects of autobiographical memory. For example, in the recent study of Brown et al. (2018), participants were asked to judge the temporal sequence of past events from their own life or lives of others. While activity in most of the MTL regions was related to EAM, neural activity in superior parietal lobule, intraparietal sulcus and hippocampal tail showed similar involvement for both episodic and semantic autobiographical memories. Importantly, activity in the AG revealed a graded pattern from episodic recollection to semantic remembering. Furthermore, several neuroimaging studies (Addis et al., 2004a,b; Levine et al., 2004; Holland et al., 2011) showed enhanced activity in the left parahippocampus, left temporoparietal junction, fusiform gyri and right inferior temporal cortex when past events are repeated compared to unique events. Recently, Jonker et al. (2018) developed a paradigm during which participants encoded object-scene pairs, following with retrieval resulting in increased re-activation of both target objects and contextually related objects in a network including the hippocampus and posterior medial network of parietal regions, known as the DMN (Ranganath and Ritchey, 2012; Rugg and Vilberg, 2013). Based on the present meta-analytical findings we suggest that BSC-related processing in bilateral AG may play an important role in EAM, especially with respect to subjective re-experiencing of EAM. However, it is important to highlight that this anatomical overlap between EAM and BSC is small and both cognitive functions have a largely separated basis. Furthermore, an important limitation of the present study is we could only include a single neuroimaging study that experimentally investigated self-location and first-person perspective. Future work will hopefully be able to perform meta-analysis also for these BSC components, thereby improving the analysis with respect to EAM, as well as with other forms of perspective manipulations relying on viewpoint changes or mental imagery.

## Conclusion

Recent data in cognitive neuroscience suggest that the lateral parietal cortex contributes to the EAM retrieval. The present study extends earlier neuroimaging and neuropsychological patient work and provides preliminary evidence for shared neural parietal resources between BSC and EAM, but not SAM. The present data suggest that conscious re-experiencing of past life episodes (EAM) and the conscious on-line experience of being located and experiencing the world first-person (BSC) both depend on the lateral parietal cortex structures. We find that especially the AG is jointly involved bilaterally in processing related to BSC and EAM and may be a key structure for neural processing related to self-consciousness including conscious online experiencing and later re-experiencing in EAM.

## Author Contributions

LB, PG and OB contributed to the conception and design of the study. LB organized the meta-analytical database and wrote the first draft of the manuscript. LB and PG performed the statistical analysis. OB wrote sections of the manuscript. All authors contributed to manuscript revision, read and approved the submitted version.

## Conflict of Interest Statement

The authors declare that the research was conducted in the absence of any commercial or financial relationships that could be construed as a potential conflict of interest.

## Footnotes

^1^https://www.ncbi.nlm.nih.gov/pubmed/

^2^http://apps.webofknowledge.com/WOS_GeneralSearch_input.do?product=WOS&search_mode=GeneralSearch&SID=E1ZUdnq9Nmid1G5uAnw&preferencesSaved=

^3^http://people.cas.sc.edu/rorden/mricron/index.html
