# Supplementary material for: Common Recruitment of Angular Gyrus in Episodic Autobiographical Memory and Bodily Self-Consciousness
Source: Front Behav Neurosci. 2018 Nov 14;12:270. doi: 10.3389/fnbeh.2018.00270 (PMC6246737; doi:10.3389/fnbeh.2018.00270)
Supplement: Supplementary file 1 [file Data_Sheet_1.docx]

| **Reference** | **Contrast** | **Imaging method** | **Number of foci** | **Number of participants** |
| --- | --- | --- | --- | --- |
| Addis et al. (2004a) | specific and generic EAM vs semantic control task | fMRI | 16 | 14 |
| Addis et al. (2004b) | specific and generic EAM vs semantic control task | fMRI | 54 | 14 |
| Addis et al. (2012) | EAM vs semantic and visuospatial control tasks | fMRI | 18 | 15 |
| Botzung et al. (2008b) | EAM vs SM | fMRI | 34 | 10 |
| Cabeza et al. (2004) | EAM and lab retrievals | fMRI | 10 | 13 |
| Conway et al. (1999) | recent and remote EAM vs cued recall of paired associates | PET | 11 | 6 |
| Daselaar et al. (2008) | EAM access and elaboration | fMRI | 20 | 17 |
| Denkova et al. (2006a) | EAM vs SM | fMRI | 26 | 10 |
| Denkova et al. (2006b) | EAM vs SM | fMRI | 25 | 20 |
| Donix et al. (2010b) | recent and remote EAM vs recent and remote SM | fMRI | 26 | 15 |
| Fink et al. (1996) | EAM vs rest and impersonal task | fMRI | 9 | 7 |
| Gardini et al. (2006) | EAM vs baseline | fMRI | 20 | 14 |
| Gilboa et al (2004) | EAM vs semnatic control task | fMRI | 22 | 9 |
| Greenberg et al. (2005) | EAM vs semantic control task | fMRI | 18 | 16 |
| Hennessey Ford et al. (2011) | EAM vs semantic control task | fMRI | 21 | 16 |
| Holland et al. (2011) | EAM vs semantic control task | fMRI | 9 | 31 |
| Hoscheidt et al. (2010) | EAM vs description of music with adjectives | fMRI | 27 | 17 |
| Levine et al (2004) | personal episodic memories vs personal semantic | fMRI | 20 | 5 |
| Maguire et al. (1999) | personal episodic memories vs non personal memories | PET | 9 | 8 |
| Maguire et al. (2003a) | EAM vs control task | fMRI | 10 | 24 |
| Maguire et al. (2003b) | personal episodic memory vs public event control task | fMRI | 6 | 24 |
| Mayes et al. (2004) | EAM vs semantic control task | fMRI | 14 | 9 |
| Milton et al. (2011) | remember vs new responses | fMRI | 36 | 15 |
| Nadel et al. (2007) | remote and recent EAM vs rest | fMRI | 16 | 12 |
| Oddo et al. (2010) | remote and recent EAM vs SM | fMRI | 14 | 15 |
| Okuda et al. (2003) | EAM vs baseline | fMRI | 18 | 12 |
| Piefke et al. (2003) | EAM vs baseline | fMRI | 18 | 20 |
| Piolino et al. (2008) | EAM vs semantic control | fMRI | 19 | 12 |
| Rabin et al. (2009) | vivid EAM and Tom vs baseline | fMRI | 20 | 20 |
| Ryan et al. (2001) | EAM vs semantic control task | fMRI | 22 | 7 |
| Söderlung et al. (2012) | EAM vs odd/even number judgement | fMRI | 19 | 12 |
| St Jacques et al. (2011) | EAM access and elaboration | fMRI | 28 | 17 |
| St Jacques et al. (2011) | EAM access and elaboration | fMRI | 15 | 17 |
| St Jacques et al. (2012) | self projection of one's own self | fMRI | 13 | 23 |
| Summerfield et al. (2009) | real EAM vs imagined EAM | fMRI | 12 | 18 |
| Svoboda et al. (2009) | EAM vs general semantic memory | fMRI | 19 | 11 |
| Trinkler et al. (2009) | recognition task | fMRI | 25 | 14 |
| Tsukuira et al. (2002) | recall of personal events vs retrieval of lexical meaning of words | fMRI | 26 | 9 |
| Vanderkerchove et al. (2005) | EAM vs rest | fMRI | 16 | 16 |
| Viard et al. (2007) | EAM vs low level baseline conditions (i.e. pseudowords) | fMRI | 24 | 12 |
| Viard et al. (2011) | EAM vs low level baseline conditions (i.e. pseudowords) | fMRI | 28 | 12 |

**Supplementary Table 1.** Overview of studies investigating neural correlates of episodic autobiographical memory (EAM).

fMRI= functional magnetic resonance

PET= positron emission tomography

| **Reference** | **Contrast** | **Imaging method** | **Number of foci** | **Number of participants** |
| --- | --- | --- | --- | --- |
| Addis et al. (2004a) | SAM vs EAM | fMRI | 3 | 14 |
| Donix et al. (2010a) | familiar faces + places vs unknown faces and places | fMRI | 16 | 12 |
| Gobbini et al. (2004) | familiar faces vs unfamiliar faces | fMRI | 19 | 10 |
| Leibenluft et al (2004) | familiar faces vs unfamiliar faces | fMRI | 36 | 7 |
| Levine et al. (2004) | SAM vs EAM | fMRI | 21 | 5 |
| Maddock et al. (2001) | familiar names vs unfamiliar names | fMRI | 24 | 8 |
| Maguire et al. (2003a) | SAM vs control | fMRI | 17 | 12 |
| Nakamura et al. (2000) | (familiar faces - control) vs familiar places - control) | PET | 6 | 7 |
| Shah et al (2001) | familiar faces and voices vs unfamiliar faces and voices | fMRI | 2 | 10 |
| Sugiura et al. (2005) | familiar objects and places vs unfamiliar objects | fMRI | 7 | 25 |
| Suguira et al (2006) | familiar names vs unfamiliar names | fMRI | 47 | 24 |
| Suguira et al. (2009) | familiar names vs unfamiliar names | fMRI | 7 | 28 |
| Suguira et al. (2011) | familiar faces vs unfamiliar faces | fMRI | 9 | 24 |
| D'argembeau et al. (2008) | self trait judgement vs other traits judgement | fMRI | 8 | 16 |
| Gutchess et al. (2007) | self trait judgement vs other traits judgement | fMRI | 9 | 19 |
| Heatherton et al. (2006) | self trait judgement vs other traits judgement | fMRI | 9 | 30 |
| Jenkins et al. (2008) | self-opinion vs other-opinion | fMRI | 1 | 13 |
| Kelley et al. (2002) | self trait judgement vs other traits judgement | fMRI | 2 | 24 |
| Kjaer et al. (2002) | self trait judgement vs other traits judgement | PET | 16 | 7 |
| Modinos et al. (2009) | self trait judgement vs other traits judgement | fMRI | 11 | 16 |
| Oschner et al. (2005) | self trait judgement vs other traits judgement | fMRI | 6 | 16 |
| Pfeifer et al. (2007) | self trait judgement vs other traits judgement | fMRI | 25 | 12 |
| Schmitz et al. (2004) | self trait judgement vs other traits judgement | fMRI | 6 | 19 |
| Seger et al. (2004) | self trait judgement vs other traits judgement | fMRI | 4 | 12 |
| Zhu et al. (2007) | self trait judgement vs other traits judgement | fMRI | 3 | 26 |

**Supplementary Table 2.** Overview of studies investigating neural correlates of semantic autobiographical memory (SAM).

fMRI= functional magnetic resonance

PET= positron emission tomography
